# Supercharged NK cells: a unique population of NK cells capable of differentiating stem cells and lysis of MHC class I high differentiated tumors

**DOI:** 10.1038/s41419-025-07986-2

**Published:** 2025-09-01

**Authors:** Kawaljit Kaur, Po-Chun Chen, Anahid Jewett

**Affiliations:** 1 Division of Oral Biology and Medicine, The Jane and Jerry Weintraub Center for Reconstructive Biotechnology, University of California School of Dentistry, Los Angeles, USA; 2 The Jonsson Comprehensive Cancer Center, UCLA, Los Angeles, CA USA

**Keywords:** Oral cancer, Cancer immunotherapy

## Abstract

This study highlights the significance of supercharged NK (sNK) cells in inducing the lysis and differentiation of tumors at much higher levels compared to primary activated NK cells. sNK cells-induced higher release of growth factors, cytokines, and chemokines when compared to primary activated NK cells. When we used a similar level of IFN-γ from primary activated NK cells and sNK cells, the IFN-γ secreted from sNK cells exhibited greater potential to induce differentiation in both oral and pancreatic tumors. It is long known in the field of NK cells that primary NK cells induce significant lysis of stem-like/poorly differentiated tumors, but differentiated tumors are generally resistant to primary NK cell-mediated lysis. sNK cells, unlike primary activated NK cells, are found to highly target stem-like as well as differentiated tumors, indicating sNK cells can target not only tumors specific to NK cells but also those targeted by CD8+ T cells. Differentiation by sNK cells was inhibited less by the antibodies to IFN-γ and TNF-α when compared to that mediated by the primary activated NK cells, suggesting the role of other unexplored mechanisms in sNK cell-induced tumor differentiation. Overall, this study suggests the role of sNK cells in targeting the heterogeneous population of tumors, likely mediating the functions of both NK cells and T cells in controlling tumors, and inducing them to be effectively targeted by chemotherapy.

## Introduction

Natural killer (NK) cells were found to play a significant role in recognizing, lysing, and differentiating several cancer stem-like cells (CSCs)/poorly differentiated tumors [1–4]. NK cell-based cancer immunotherapies are found to be effective against several tumors [5–10]. Even though NK cells are a great tool to treat cancer, there are many hurdles, such as a minimal (5–15% of total peripheral blood mononuclear cells) number of NK cells in peripheral blood, dysfunctional endogenous NK cells in cancer patients, the short lifespan of NK cells in vivo (approximately 7 days), mechanisms contributing to reduced NK cell infiltration to the tumor site, particularly in solid tumors, and functional impairments in the tumor microenvironment [11–14].

**Fig. 1:**
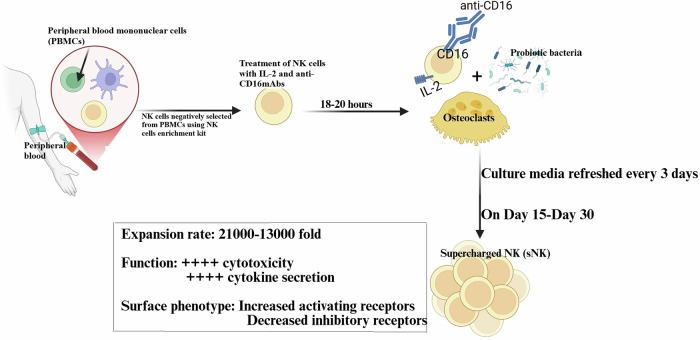
Illustration to show the generation process of supercharged NK cells. Human peripheral blood mononuclear-derived NK cells were activated with rh-IL-2 (1000 U/ml) and anti-CD16 mAb (3 µg/ml) for 18–20 h before they were co-cultured with OCs and probiotic bacteria sAJ2 (OCs:NK:sAJ2; 1:2:4). The medium was refreshed every three days with RPMI containing rh-IL-2 (1500 U/ml).

To find the solution to above mentioned problems, our laboratory has developed a technology to upregulate the number and functional activation of NK cells by co-culturing primary NK cells with osteoclasts (OCs) as feeder cells in the presence of probiotics (Fig. 1) [4, 15, 16]. Previous studies from our laboratory have demonstrated that OCs express increased surface expression of ligands for NK cell surface activating surface receptors, such as MICA/B, KLRG1, and ULBPs, and OC-secreted cytokines and chemokines, such as IL-12, IL-15, IFN-γ, and IL-18, play a significant role in NK cell activation [17, 18]. Collaborative effects of these OC ligands and secreted factors result in increased cell expansion and functional activation of NK cells during OCs and NK cells’ cell-to-cell contact. Treatment of NK cells with a combination of seven gram-positive probiotic bacteria strains (*Streptococcus thermophiles*, *Bifidobacterium longum*, *Bifidobacterium breve*, *Bifidobacterium infantis*, *Lactobacillus acidophilus*, *Lactobacillus plantarum*, and *Lactobacillus paracasei)* was found to increase cytokine secretion by NK cells, including IFN-γ, which could further facilitate signals required for NK cell expansion [4, 19–22]. Thus, the main goal of using OCs and probiotic bacteria is to induce signals participating in increased lifespan, cell expansion, and functional activation in NK cells [4, 15, 21]. Due to their superior anti-cancer activity in vitro and in vivo, these cells were named supercharged NK (sNK) cells [2–4, 15, 16, 20, 21, 23–37]. sNK cells were found to be highly effective against tumors in preclinical models [2–4, 15, 16, 20, 21, 23–37].

Several characteristic differences between CSCs and differentiated tumors have been demonstrated based on surface expression, susceptibility to NK cell-mediated cytotoxicity, therapeutic response, growth profile, metastasis, and clonogenicity, etc. CSCs, compared to differentiated tumors found to express lower levels of surface EGF-R, CD166, MHC class I, CD54, and PD-L1 (B7H1) receptors and higher expression of CD133, CD44, EpCAM, CD26, and CD338 [1–3, 19, 38–41]. CSCs express higher susceptibility to NK cell-mediated cytotoxicity compared to differentiated tumors [1, 3, 19, 39, 41]. CSCs were found to be more aggressive, contributing to increased tumor cell growth, migration, clonogenicity, self-renewal capacity, chemotherapy resistance, radiotherapy resistance, increased DNA mismatch repair, and multidrug resistance genes [1, 19, 21, 42–52]. Also, CSCs, when co-cultured with NK cells, triggered significant IFN-γ secretion by NK cells, but co-culture of differentiated tumors with NK cells triggered no or lower IFN-γ secretion by the NK cells [53]. Among all these, we observed a consistent relationship between the differentiation stage of the tumors and their susceptibility to primary NK cell-mediated cytotoxicity [1, 3, 19], and lower surface of MHC class I and CD54 expression, whereas well-differentiated tumors exhibit much higher expression of these surface molecules [1, 19, 21]. Notably, stem-like tumors with lower MHC class I and CD54 expression were more susceptible to primary NK cell-mediated killing than their well-differentiated counterparts [1, 19, 21]. Based on these findings, in this study, we focused on three criteria to demonstrate differentiation in tumors: 1. Surface expression of MHC class I and CD54 [1, 3, 19]; 2. Level of primary NK cell-mediated lysis against tumors [1, 19, 21]; 3. Level of chemo-drug-induced lysis against tumors [1]. IFN-γ and TNF-α secreted by NK cells were found to play a key role in NK cell-induced differentiation in tumors, and differentiation in tumors increases their sensitivity to chemotherapy drugs [1, 54].

Findings of this study extend our previous work on sNK cells. First, in addition to IFN-γ and TNF-α, sNK cells were found to secrete higher levels of several other factors playing a role in anti-cancer activity and activating other immune cells. Secondly, studies performed in which primary NK cells and sNK cells were used as effectors against stem-like and differentiated tumors revealed the potential of sNK cells to lyse both stem-like and differentiated tumors, whereas primary NK cells only lysed stem-like tumors. Third, sNK cells were found to be highly potent in inducing differentiation in tumors compared to primary NK cells. Finally, sNK cells-differentiated tumors were found to be highly susceptible to chemotherapeutic drug-induced tumor lysis compared to those differentiated by primary NK cells. Together, our results highlight distinct functional differences between primary NK cells and sNK cells in direct killing and differentiation of tumors.

## Results

### Methodology and phenotypic characteristics of supercharged NK cells

**Fig. 2:**
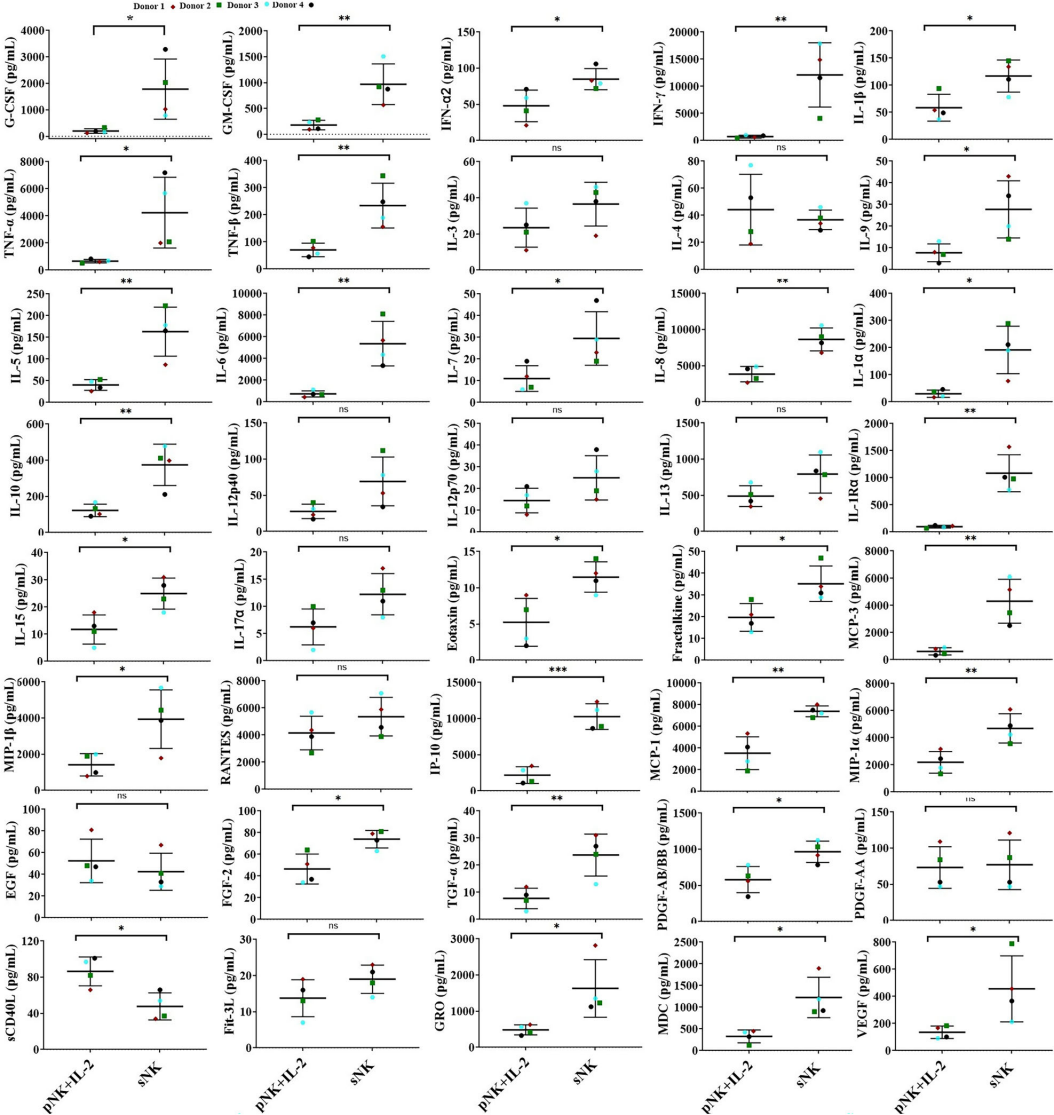
sNK cells secreted much higher levels of cytokines, chemokines, and growth factors compared to primary NK cells. OCs were generated as described in “Materials and methods” section. NK cells (1 × 10^6^ cells/ml) from healthy individuals were treated with a combination of IL-2 (1000 U/ml) and anti-CD16 mAb (3 µg/ml) for 18 h before they were cultured with the healthy individuals’ OCs and sAJ2 at a ratio of 1:2:4 (OCs:NK:sAJ2). On day 15 of sNK cell cultures, another set of NK cells was isolated from the same healthy individuals and were treated with IL-2 (1000 U/ml). The supernatants were harvested from primary IL-2-treated NK cells and sNK cells to determine secretion levels of cytokines, chemokines, and growth factors using a multiplex assay (*n* = 4).

We have previously demonstrated the detailed description of the supercharged NK (sNK) cells generation process and their superior cell expansion and anti-cancer activity [4, 15, 20, 21, 23, 55]. Briefly, NK cells are isolated from peripheral blood-derived mononuclear cells (PBMCs) and are treated with recombinant human IL-2 (rh-IL-2) and anti-CD16 mAbs overnight. After which, activated NK cells are co-cultured with osteoclasts and sonicated probiotic AJ2 bacteria (sAJ2) for 15–30 days (Fig. 1). Supercharged NK cells were found to exhibit significantly increased survival, increased cell expansion, increased cytokine secretions, increased cytotoxic activity, increased expression of activating surface receptors, and decreased expression of inhibitory receptors compared to primary NK cells [4, 17, 55] (Fig. 1). In this study, we further characterized sNK cells using a multiplex assay and demonstrated increased secretion levels of cytokines, chemokines, and growth factors of sNK cells compared to primary IL-2-treated NK cells, except IL-4, EFG, PDGF-11, and sCD40L (Fig. 2 and Table 1).

**Table 1:** sNK cells secreted much higher levels of cytokines, chemokines, and growth factors compared to primary NK cells.

Factors	OCs + Probiotics (pg/ml: mean ± std dev.)	pNK + IL-2 (pg/ml: mean ± std dev.)	sNK (pg/ml: mean ± std dev.)	sNK (pg/ml) /pNK + IL-2 (pg/ml) fold change mean ± std dev.
**G-CSF**	29 ± 7	198 ± 88	1784 ± 1140	9.3 ± 5.6
**GM-CSF**	84 ± 13	179 ± 91	967 ± 393	6 ± 2
**IFN-a2**	NA	48 ± 22	85 ± 15	2.2 ± 1.2
**IFN-γ**	0	731 ± 264	12109 ± 5938	16.5 ± 7.4
**IL-1β**	0	56 ± 25	117 ± 27	2.1 ± 0.4
**TNF-α**	23 ± 3	664 ± 131	4239 ± 2608	6 ± 2.8
**TNF-β**	NA	71 ± 25	234 ± 82	4 ± 1.4
**IL-3**	NA	24 ± 11	37 ± 12	1.6 ± 0.3
**IL-4**	2 ± 0.9	44 ± 26	37 ± 7	1.1 ± 0.6
**IL-9**	8 ± 4	8 ± 4	28 ± 13	5.1 ± 4.5
**IL-5**	0	40 ± 12	163 ± 56	4 ± 0.6
**IL-6**	39 ± 11	739 ± 282	5372 ± 2060	8.3 ± 4.6
**IL-7**	0	11 ± 6	30 ± 12	3 ± 1.2
**IL-8**	3891 ± 123	3842 ± 1059	8638 ± 1577	2.3 ± 0.4
**IL-1a**	NA	30 ± 13	192 ± 87	6.5 ± 2.3
**IL-10**	12 ± 5	123 ± 34	375 ± 114	3.1 ± 0.6
**IL-12p40**	NA	28 ± 10	70 ± 34	2.4 ± 0.3
**IL-12p70**	9 ± 3	15 ± 6	25 ± 10	2 ± 0.13
**IL-13**	0	489 ± 143	795 ± 264	1.6 ± 0.3
**IL-1Ra**	89 ± 12	97 ± 24	1085 ± 340	11.5 ± 3.5
**IL-15**	34 ± 9	12 ± 5	25 ± 6	2.4 ± 0.8
**IL-17a**	29 ± 16	6.3 ± 3.3	12.3 ± 3.7	2.4 ± 1.3
**Eotaxin**	0	5.3 ± 3.3	12 ± 2	3 ± 1.8
**Fractalkine**	NA	20 ± 6.4	36 ± 8	2 ± 0.2
**MCP-3**	NA	619 ± 267	4314 ± 1623	7.2 ± 0.5
**MIP-1β**	167 ± 33	1417 ± 618	3041 ± 1609	2.8 ± 0.76
**RANTES**	1789 ± 234	4152 ± 1236	5360 ± 1421	1.3 ± 0.12
**IP-10**	1356 ± 89	2183 ± 1156	10281 ± 1790	6 ± 2
**MCP-1**	2178 ± 442	3527 ± 1510	7391 ± 506	2.4 ± 0.9
**MIP-1α**	16 ± 7	2189 ± 796	4698 ± 1079	2.3 ± 0.3
**EGF**	0	53 ± 20	42 ± 17	0.8 ± 0.07
**FGF-2**	NA	46 ± 14	74 ± 8	1.7 ± 0.3
**TGF-α**	27 ± 9	8 ± 4	24 ± 8	3.3 ± 0.7
**PDGF-AB/BB**	123 ± 17	580 ± 181	965 ± 147	1.7 ± 0.4
**PDGF-AA**	NA	73 ± 29	77 ± 34	1.04 ± 0.05
**sCD40L**	NA	87 ± 16	48 ± 15	0.5 ± 0.08
**Fit-3L**	NA	14 ± 5	19 ± 4	1.5 ± 0.3
**GRO**	NA	484 ± 139	1632 ± 797	3.3 ± 0.8
**MDC**	NA	329 ± 147	1226 ± 465	4.3 ± 2
**VEGF**	67 ± 8	135 ± 46	456 ± 244	3.3 ± 0.8

OCs were generated as described in “Materials and methods” section. NK cells (1 × 10^6^ cells/ml) from healthy individuals were treated with a combination of IL-2 (1000 U/ml) and anti-CD16 mAb (3 µg/ml) for 18 h before they were cultured with the healthy individuals’ OCs and sAJ2 at a ratio of 1:2:4 (OCs:NK:sAJ2). On day 15 of sNK cell cultures, OCs were treated with sAJ2 (OC:sAJ2: 1:4), and another set of NK cells was isolated from the same healthy individuals and were treated with IL-2 (1000 U/ml). The supernatants were harvested from primary sAJ2-treated OCs (column 2), IL-2-treated NK cells (column 3), and sNK cells (column 4) to determine secretion levels of cytokines, chemokines, and growth factors using a multiplex assay (*n* = 4). Fold change of sNK cells vs. primary NK cells was determined by using the formula: Level of IFN-γ (pg/ml) secreted by sNK cells/ Level of IFN-γ (pg/ml) secreted by primary NK cells of the same donor (*n* = 4) (column 5).

### sNK cells mediate significant lysis in both stem-like and differentiated tumors

**Fig. 3:**
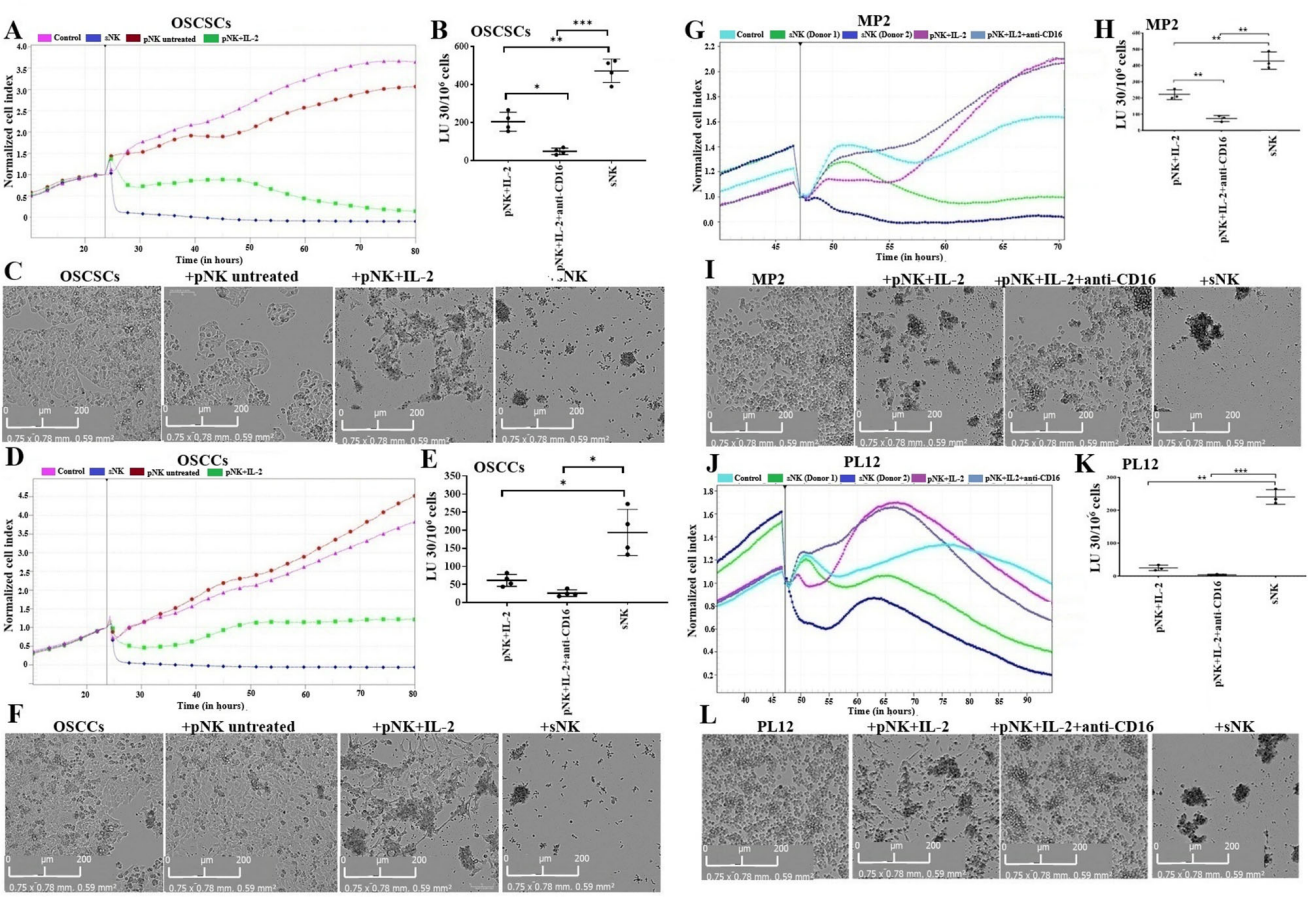
sNK cells induced a significant level of killing in both stem-like and differentiated tumors. OSCSCs (**A**, **C**), OSCCs (**D**, **F**), MP2 (**G**, **I**), and PL12 (**J**, **L**) were cultured on eSight plates for 20–24 h before primary untreated, primary activated, and sNK cells were added at 2.5:1 E:T ratio, and co-cultures were continued to 70–80 h. The graphs for normalized cell index were assessed by the eSight RTCA and RTCA pro software (**A**, **D**, **G**, **J**). Microscopic images of tumor and NK cell interactions, as shown in the figures, were captured by eSight after 24 h of incubation (scale: 0–200 µm) (**C**, **F**, **I**, **L**). sNK cells were generated as described in Fig. 1, on day 15 of sNK cell cultures, another set of NK cells was isolated from the same healthy individuals and were treated with IL-2 (5000 U/ml) and with a combination of IL-2 (5000 U/ml) and anti-CD16 mAb (3 µg/ml) for 18 h. Primary activated NK cells and sNK cells were used as effectors against OSCSCs (**B**), OSCCs (**E**), MP2 (**H**), and PL12 (**K**) to measure NK cell-mediated cytotoxicity using a standard 4-h ^51^Cr release assay against tumor cells. The lytic units (LU) 30/10^6^ cells were determined using the inverse number of NK cells required to lyse 30% of target cells × 100.

It was demonstrated before that primary NK cells induce significant lysis in CSCs, but differentiated tumors are resistant to primary NK cell-mediated cytotoxicity [1, 19, 21, 39, 40, 53, 56]. Data shown in Fig. 3 demonstrated that, unlike primary NK cells, sNK cells can induce significant lysis in both stem-like as well as differentiated oral (Fig. 3A–F) and pancreatic (Fig. 3G–L) tumors. Data obtained by a long-term killing assay using eSight showed the lowest cell index of OSCSCs (Fig. 3A), OSCCs (Fig. 3D), MP2 (Fig. 3G), and PL12 (Fig. 3J) in sNK cell cultures compared to primary untreated or activated NK cells. Microscopic images of tumor and NK cell interactions were captured by eSight after 24 h of incubation. As seen in the microscopic analysis, sNK cells-induced higher lysis in OSCSCs (Fig. 3C), OSCCs (Fig. 3F), MP2 (Fig. 3I), and PL12 (Fig. 3L) compared to primary untreated or activated NK cells. Data obtained by four-hour chromium release assay also have shown higher tumor killing by sNK cells compared to primary activated NK cells (Fig. 3B, E, H, and K). Thus, sNK cells can be the tool to eradicate heterogeneous populations of tumors.

### Enhanced tumor differentiation induced by sNK cells compared to primary NK cells

**Fig. 4:**
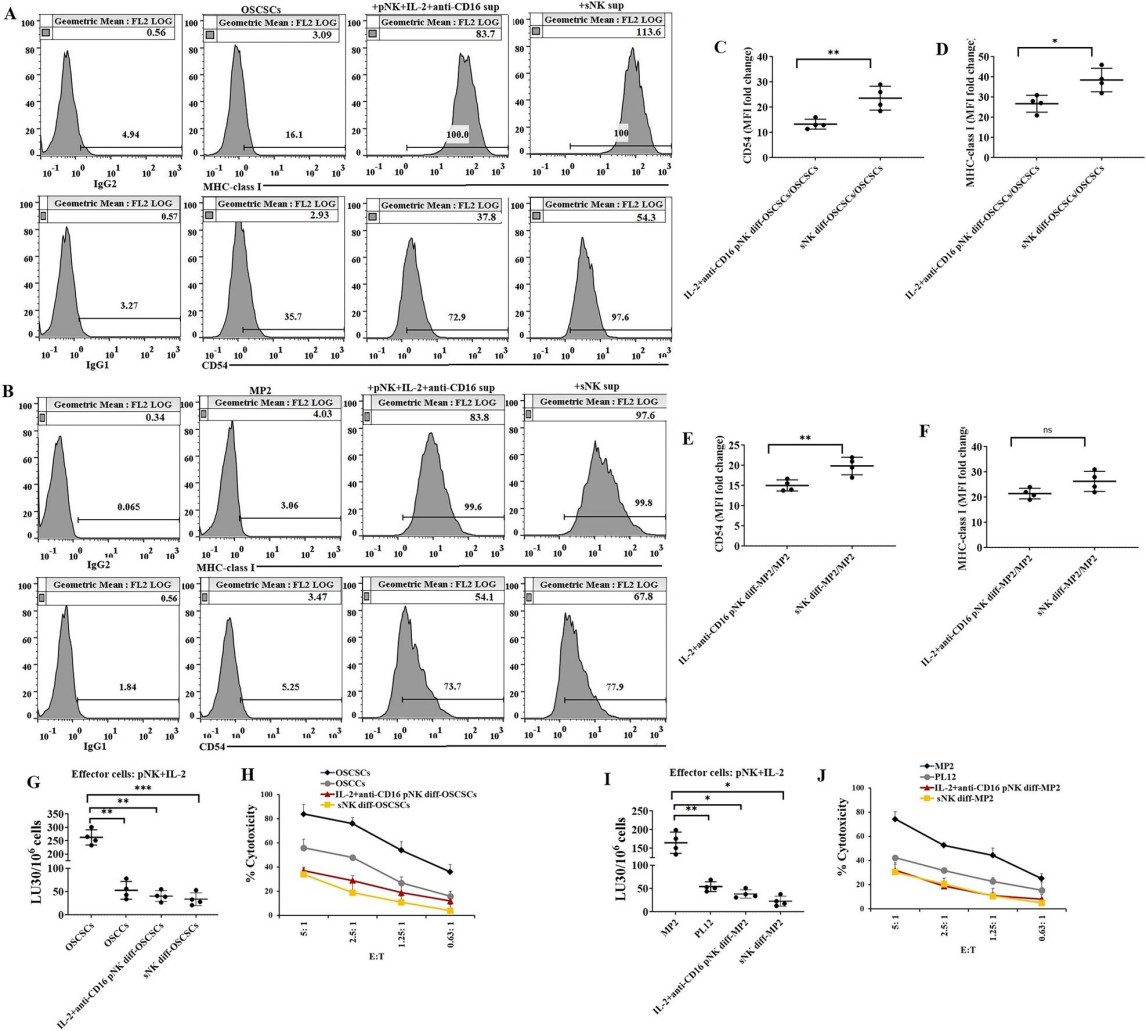
Changes in tumor cell surface proteins induced by supernatants from supercharged NK cells compared to primary NK cells. Differentiation of OSCSCs (**A**, **C**) and MP2 (**B**, **D**) was conducted with an average total of 2000 pg and 5000 pg IFN-γ. sNK cells were generated as described in Fig. 1, on day 15 of sNK cell cultures, another set of NK cells was isolated from the same healthy individuals and were treated with a combination of IL-2 (1000 U/ml) and anti-CD16 mAb (3 µg/ml) for 18 h. The supernatants were harvested from primary IL-2 and anti-CD16 mAbs-treated NK cells and sNK cells to determine IFN-γ secretion using a single ELISA. The volume of NK cell supernatants to treat the tumor cells was determined based on the levels of IFN-γ in the supernatants as detected by ELISA and was divided over 4 days with daily treatment. On day 5, the surface expression levels of MHC class I and CD54 on OSCSCs (**A**) and MP2 (**B**) were assessed using flow cytometric analysis. Isotype control IgG2 and IgG1 were used for MHC class I and CD54, respectively, and a single isotype control was used for untreated, primary NK cell-supernatant-treated, and sNK cell-supernatant-treated OSCSCs (**A**) or MP2 (**B**). Fold change in the MFI of CD54 (**C**, **E**) and MHC class I (**D**, **F**) was determined for NK cells supernatants treated vs. untreated OSCSCs and MP2, respectively, using the formula: MFI of pNK or sNK supernatant/MFI of untreated tumors (*n* = 4) (**C**–**F**). Primary NK cells were treated with IL-2 (1000 U/mL) for 18 h and were used as effectors against untreated and primary or sNK cells’ supernatant-treated OSCSCs (**G**, **H**) and MP2 (**I**, **J**) to measure NK cell-mediated cytotoxicity using a standard 4-h ^51^Cr release assay against tumor cells. OSCCs and PL12 were used as positive controls, as differentiated oral and pancreatic tumors, respectively (**G**–**J**). The lytic units (LU) 30/10^6^ cells were determined using the inverse number of NK cells required to lyse 30% of target cells × 100 (*n* = 4) (**G**, **I**). The percentage killing of the tumor by NK cells at different effector-to-target ratios is shown in the figure (**H**, **J**). One of the representative experiment data (mean ± std dev.) is shown in (**H** and **J**).

We have previously demonstrated that supernatants of NK cells mediated the differentiation of tumor CSCs, and IFN-γ secreted from NK cells plays a key role in tumor differentiation [19, 40]. Differentiated tumors exhibited increased surface expression levels of MHC class I and CD54 and decreased sensitivity to NK cell-mediated cytotoxicity [19, 40]. To compare the differentiation level in tumors by primary NK cells and sNK cells, we treated CSCs, oral squamous carcinoma stem-like cells (OSCSCs) (Fig. 4A) and pancreatic tumors MIA PaCA-2 (MP2) (Fig. 4B), with the supernatants derived from primary NK cells activated with IL-2 and anti-CD16 monoclonal antibodies (IL-2 + anti-CD16 mAbs), as primary NK cells treated with IL-2 + anti-CD16 mAbs has demonstrated higher levels of cytokine secretion compared to untreated or IL-2-treated primary NK cells [57], as well as from sNK cells. The volume of supernatant for tumor treatments was determined based on IFN-γ levels in the culture media of primary IL-2 + anti-CD16 mAbs-treated NK cells and sNK cells. Higher levels of MHC class I and CD54 surface expression were seen in sNK cell-supernatant-treated OSCSCs (Fig. 4A) and MP2 (Fig. 4B) compared to those treated with primary IL-2 + anti-CD16 mAbs-treated NK cell supernatant. When we determined the fold change of CD54 and MHC class I MFIs of NK cell-supernatant-treated tumors compared to untreated tumors, significant differences were seen between sNK cell-supernatant-treated tumors compared to primary NK cell-supernatant-treated tumors (Fig. 4C–F). Increased resistance to primary IL-2-treated NK cell-mediated cytotoxicity was also seen in sNK cell-supernatant-treated OSCSCs (Fig. 4G, H) and MP2 (Fig. 4I, J) compared to those treated with primary IL-2 + anti-CD16 mAbs-treated NK cell supernatant.

**Fig. 5:**
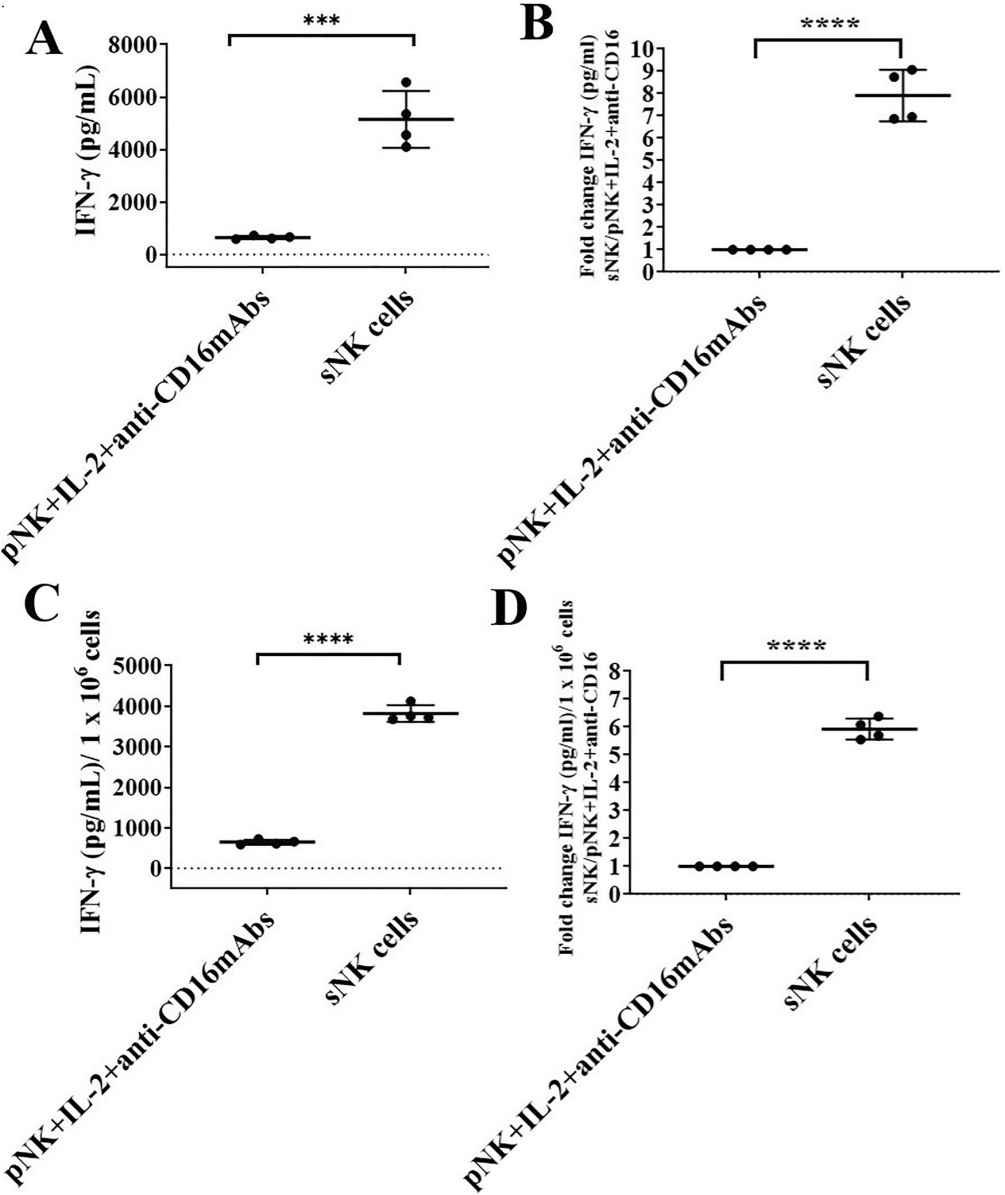
Increased levels of IFN-γ secretion by sNK cells compared to primary activated NK cells. sNK cells were generated as described in Fig. 1. On day 15 of sNK cell cultures, another set of NK cells was isolated from the same healthy individuals and were treated with a combination of IL-2 (1000 U/ml) and anti-CD16 mAb (3 µg/ml) for 18 h. The supernatants were harvested from primary IL-2 and anti-CD16 mAbs-treated NK cells and sNK cells to determine IFN-γ secretion using a single ELISA (*n* = 4) (**A**). Fold change in IFN-γ was determined by using the formula: (left bar) IFN-γ of IL-2 + anti-CD16 mAbs-treated pNK cells/ IFN-γ of IL-2 + anti-CD16 mAbs-treated pNK cells, (right bar) IFN-γ of sNK cells/IFN-γ of IL-2 + anti-CD16 mAbs (*n* = 4) (**B**). The amounts of IFN-γ secretion shown in Fig. 5A were determined based on 1 × 10^6^ cells (*n* = 4) (**C**). Fold change in IFN-γ was determined by using the formula: (left bar) IFN-γ per million cells of IL-2 + anti-CD16 mAbs-treated pNK cells/IFN-γ per million cells of IL-2+anti-CD16 mAbs-treated pNK cells, (right bar) IFN-γ per million cells of sNK cells/IFN-γ per million cells of IL-2 + anti-CD16 mAbs (*n* = 4) (**D**).

When we compared the IFN-γ secretion levels in the culture media of IL-2 + anti-CD16 mAbs-treated primary NK cells and sNK cells, we observed significantly higher IFN-γ secretion levels in sNK cells (Fig. 5A–D). As shown in Fig. 5B, D, sNK cells secrete six to nine times more IFN-γ compared to primary IL-2 + anti-CD16 mAbs-treated NK cells. Hence, we need six to nine times less supernatant from sNK cells compared to primary NK cells to induce similar levels of differentiation on both oral and pancreatic CSCs.

### Antibodies against IFN-γ and TNF-α blocked differentiation in tumors

**Fig. 6:**
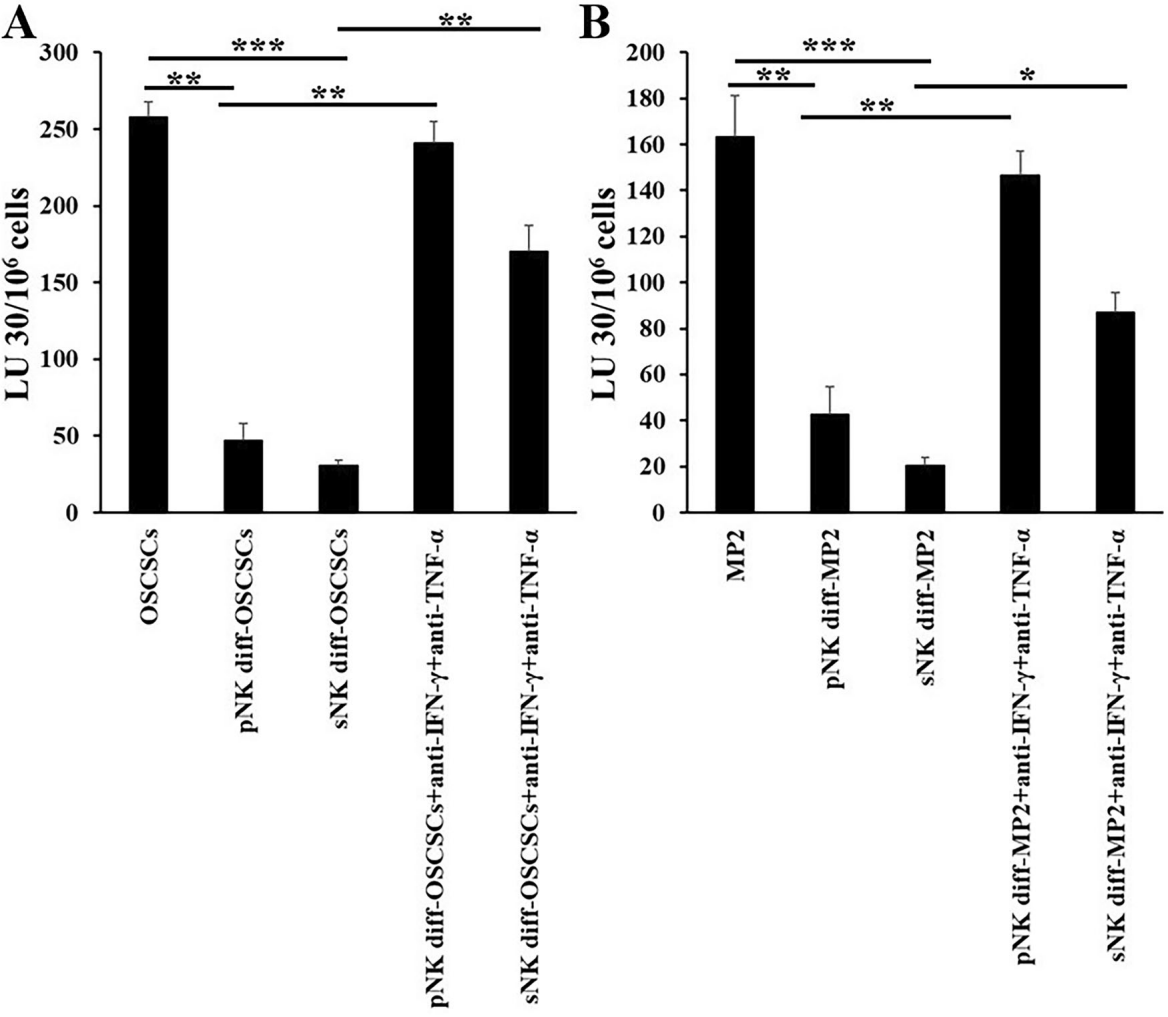
Antibodies against IFN-γ and TNF-α blocked the differentiation of tumors, an increased effect was seen in primary NK differentiated tumors compared sNK differentiated tumors. Differentiation of OSCSCs (**A**) and MP2 (**B**) was conducted as described in Fig. 4. Primary IL-2 and anti-CD16 mAbs-treated and sNK cells’ supernatant-treated tumors were a combination of anti-TNFα mAbs (1:100) and anti-IFNγ mAbs (1:100) for six days. Freshly purified NK cells were treated with IL-2 (1000 U/mL) for 18 h and were used as effectors against untreated tumors, and primary IL-2 and anti-CD16 mAbs-treated, and sNK cells’ supernatant-treated tumors in the absence or presence of anti-TNFα mAbs and anti-IFNγ mAbs to measure NK cell-mediated cytotoxicity using a standard 4-h ^51^Cr release assay against tumor cells. The lytic units (LU) 30/10^6^ cells were determined using the inverse number of NK cells required to lyse 30% of target cells × 100 (**A**, **B**).

When we treated primary NK cells or sNK cells-differentiated tumors with antibodies against IFN-γ and TNF-α, we observed higher inhibition of differentiation by antibodies in primary NK differentiated tumors compared to sNK cells-differentiated tumors (Fig. 6). Upon differentiation, both OSCSCs (Fig. 6A) and MP2 (Fig. 6B) became resistant to primary NK cell-mediated cytotoxicity, but tumors de-differentiated when they were treated with antibodies against IFN-γ and TNF-α. The level of de-differentiation was higher in primary NK cells-differentiated tumors compared to sNK cells-differentiated tumors (Fig. 6), indicating there may be more mechanisms involved in NK cells-induced tumor differentiation.

### Increased efficacy of chemo-drugs against differentiated tumors compared to stem-like tumors

**Fig. 7:**
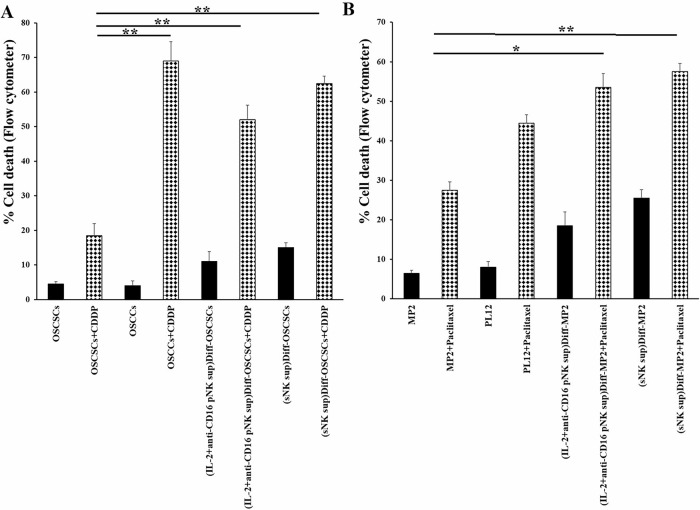
Chemotherapeutic drugs induced higher killing in differentiated tumors. Differentiation of OSCSCs (**A**) and MP2 (**B**) was conducted with an average total of 2000 pg and 5000 pg IFN-γ as described in Fig. 4. OSCSCs, OSCCs, primary NK cell-supernatant-treated OSCSCs, and sNK cell-supernatant-treated OSCSCs were treated with cisplatin (60 µg/mL) for 18–20 h, after which, the cells were stained with propidium iodide (PI) to determine percent cell death using flow cytometric analysis (**A**). MP2, PL12, primary NK cell-supernatant-treated MP2, and sNK cell-supernatant-treated MP2 were treated with paclitaxel (40 µg/mL) for 18–20 h, after which, the cells were stained with PI to determine percent cell death using flow cytometric analysis (**B**).

Differentiated tumors compared to CSCs were found to be more sensitive to chemotherapeutic drugs [1]. In this study, we induced differentiation in OSCCs and MP2 tumors using the supernatants of primary IL-2 + anti-CD16-treated NK cells and sNK cells, and determined the efficacy of chemo-drugs to induce killing in those tumors (Fig. 7). We have observed higher tumor cell death induced by CDDP (Fig. 7A) and paclitaxel (Fig. 7B) in differentiated tumors in comparison to their stem-like counterparts. Slightly higher levels of chemo-drug-induced lysis were seen in tumors differentiated by sNK cells compared to those differentiated by primary NK cells (Fig. 7).

## Discussion

Natural Killer cells target tumor cells via two different mechanisms. One by direct killing of the tumors via secreted granules, as well as through death-inducing ligands such as Fas, TNF-a, and Trail [58]. The second emerging mechanism is through differentiation of the tumors by key secreted cytokines such as IFN-γ and TNF-α [19]. The second mechanism is less studied in the field, but we have delineated the key steps in the differentiation of the tumors by the NK cells in several projects [1, 19, 59]. This mechanism not only inhibits the proliferation of less differentiated tumors, but it also allows expression of surface receptors that are associated with tumors with a differentiated phenotype [1, 19, 59]. In addition to IFN-γ and TNF-α, NK cells secrete several other cytokines, chemokines, and growth factors. These cytokines further activate macrophages to clear senescent cells [60], and also, NK cells secrete cytokines play a crucial role in modulating adaptive immunity [61–63]. When NK cells are further activated, NK cells secrete cytokines and chemokines induce regulatory activity and have a positive impact on the function of B cells and T cells [64–66], leading to cell-mediated immune response against infection, cancer, and other diseases.

In our previous work, we used oral cancer, pancreatic cancer, lung cancer, and glioblastoma to demonstrate that supernatant of NK cells mediates differentiation in tumors, and we have validated this based on IFN-γ and TNF-α levels [19, 40, 59]. In those studies, we have demonstrated the role of IFN-γ in inducing tumor differentiation. Therefore, the purpose of treating oral and pancreatic cancer stem-like cells with the supernatants of primary NK and sNK in the current study was to determine if sNK cells are more potent in mediating differentiation in tumors. We previously used CD44, MHC class I, CD54, and PD-L1 biomarkers to differentiate between less differentiated and well-differentiated tumors [1, 19, 59]. Tumors that are less differentiated express higher levels of CD44, and lower levels of MHC class I, CD54, and PD-L1; however well well-differentiated tumors express lower surface expression of CD44 but higher levels of MHC class I, CD54, and PD-L1 [1, 19, 59]. Using these criteria, we compared the ability of primary activated NK cells with sNK cells to induce differentiation in tumors. Due to increased levels of IFN-γ and TNF-α secretion by sNK cells, these cells have a higher capacity to induce differentiation in tumor cells. Even when the supernatants containing the same amount of IFN-γ from the primary activated NK cells and sNK cells were used, the supernatants from sNK cells had a better differentiation potential than primary activated NK cells. In terms of differentiation biomarkers, sNK cells had a higher increase in the expression of MHC class I and CD54 in both MP2 and OSCSC tumor lines as compared to primary activated NK cells using the same amount of IFN-γ measured in the supernatants (Fig. 4). This could be due to a synergistic effect of other factors yet to be identified in sNK cells. Similarly, we could see lower levels of primary IL-2 activated NK cell-mediated lysis of both OSCSCs and MP2 cells for sNK treated tumor cells when compared to primary activated NK cells (Fig. 4C, D). We have shown previously that when tumor cells are treated with supernatants of NK cells, they upregulate MHC class I and result in the resistance of the cells to primary NK cell-mediated cytotoxicity [1, 19, 59]. However, sNK cells are capable of targeting differentiated tumors with higher expression of MHC class I (Fig. 4) when compared to primary activated NK cells. This could be due to decreased levels of inhibitory receptors such as NKG2A on sNK cells (manuscript in press) [67]. Alternatively, or in addition, there may be other factors that synergize with the granule release or death-inducing ligands to increase the levels of tumor lysis by sNK cells. Thus, sNK cells, similar to CD8+ T cells, have acquired the ability to target MHC class I high tumor cells. Whether sNK cells can target MHC class I positive cells via peptide-mediated lysis, similar to T cells, awaits future investigation. Therefore, sNK cells have the ability to not only target NK-specific tumors (MHC class I low), but they can also lyse MHC class I high tumor cells, which are targets of CD8+ T cells.

When assessing the effect of IFN-γ and TNF-α in the differentiation of tumor cells, we used antibody blocking experiments in which both primary NK cells’ and sNK cells’ supernatant-treated tumor cells were cultured in the presence of antibodies against IFN-γ and TNF-α, and assessed the levels of differentiation. Tumors differentiated using the supernatant from primary NK cells had almost complete reversal of resistance to NK cell-mediated cytotoxicity, which is induced due to differentiation in tumors (Fig. 6). However, only 60% reversal of resistance to cytotoxicity was observed in sNK cells’ differentiated tumors, indicating that other factors may be important in causing tumor differentiation when tumor cells are treated with supernatants from sNK cells (Fig. 6). Indeed, sNK cells induce higher release of growth factors, cytokines and chemokines when compared to primary activated NK cells (Fig. 2 and Table 1).

Differentiated tumors are more susceptible to chemotherapeutic drugs [1, 59]. We next determined whether there would be differences in the primary NK cells’ and sNK cells’ supernatant-treated tumor cells in response to chemotherapeutic drugs. Using the same amounts of IFN-γ to treat the tumor cells by the primary activated NK cells’ and sNK cells’ supernatants, we observe a better synergistic lysis of the tumor cells by the sNK cells’ supernatants than those of the primary activated NK cells (Fig. 7). These results indicated that the same amount of IFN-γ and TNF-α secreted from sNK cells have better effect on tumor differentiation and lysis by the chemotherapeutic drugs than those secreted from the primary activated NK cells.

**Fig. 8:**
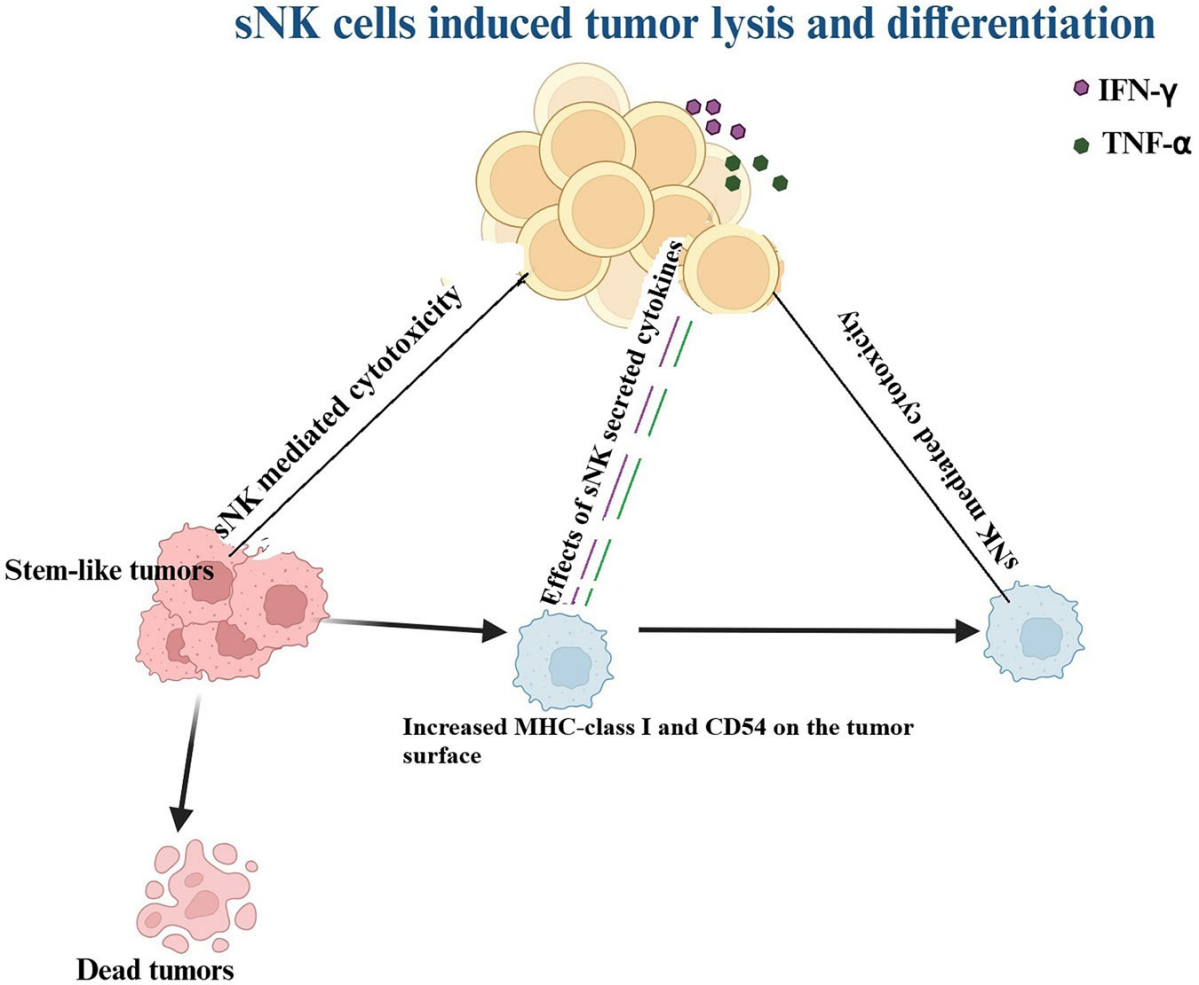
Illustration to show the efficacy of sNK cells targeting and differentiating tumors. sNK cells induce direct lysis of stem-like tumors, and differentiate remaining tumors via IFN-γ and TNF-α, and those differentiated tumors will be lysed by sNK cells. This process results in the complete eradication of the tumor.

Overall, these results suggested that sNK cells are more potent in the lysis of the tumors as well as their differentiation, and can be used in better targeting of the resistant tumors (Fig. 8). Since sNK cells carry out both NK-like functions as well as T cell functions, these cells could be categorized as a unique population of cells with both NK and T cell functions, with the ability to target all the heterogeneous clones of the tumor cells. Whether sNK cells have memory like T cells awaits future investigations. Indeed, sNK cells express higher levels of NKG2C as well as NKG2D (manuscript in press) [67].

## Materials and methods

### Cell lines, reagents, and antibodies

Oral squamous carcinoma stem cells (OSCSCs) and oral squamous cell carcinoma (OSCCs) were isolated from patients with tongue tumors at UCLA [19, 39, 40, 68]. MIA PaCA-2 (MP2) and PL12 pancreatic cancer cell lines were provided by Dr. Nicholas Cacalano (UCLA, School of Medicine, CA, USA) and were cultured in DMEM supplemented with 10% FBS. We have selected oral (OSCSCs and OSCCs) and pancreatic (MP2 and PL12) tumor cells lines because we have characterized these cells fully regarding their differentiation status and ability of NK cells to differentiate these tumors in many previous publications, and we had accumulated a lot of information regarding these cells [19, 41, 53]. These tumors grow very fast and are highly susceptible to NK cell-mediated cytotoxic effects as well as they express increased expression on CD54 and MHC class I upon treatment with NK cells’ supernatants [19]. NK cells, OSCSCs, and OSCCs were cultured in RPMI 1640 (Invitrogen by Life Technologies, CA), supplemented with 10% fetal bovine serum (FBS) (Gemini Bio-Products, CA). Recombinant IL-2 (Hoffmann-La Roche) was obtained from the NIH-BRB, National Institute of Health-Biometric Research Branch. Antibodies to CD16 (FcyRIII) (clone: 3GB) were purchased from Biolegend (San Diego, CA). Antibodies used for flow cytometry – IgG2 (clone 27-35), MHC class I (clone BB7.2), and CD54 (clone HA58) were purchased from Biolegend (San Diego, CA). Polyclonal antibodies to IFN-γ were prepared in rabbits, and monoclonal antibodies to TNF-α were prepared from the ascites of mice injected with TNF-α hybriomas. Both IFN-γ and TNF-α antibodies were purified and specificity determined by both ELISA and functional assay against recombinant IFN-γ and TNF-α, respectively. 1:100 dilution of IFN-γ and TNF-α antibodies was found to be the optimal concentration to block recombinant IFN-γ and TNF-α function. Cisplatin and paclitaxel were purchased from Ronald Reagan Pharmacy at UCLA. ELISA kits for IFN-γ were purchased from Biolegend (San Diego, CA), and a multiplex analysis kit was purchased from Millipore (Billerica, MA). Propidium iodide (PI) was purchased from PeproTech (Cranbury, NJ, USA). Chromium-51 was purchased from PerkinElmer (Springfield, IL, USA).

### Purification of human NK cells and monocytes

Written informed consents, approved by the UCLA Institutional Review Board (IRB), were obtained from healthy individuals, and all procedures were approved by the UCLA-IRB. Peripheral blood was separated using ficoll-hypaque centrifugation, after which the white, cloudy layer, containing peripheral blood mononuclear cells (PBMCs), was harvested. NK cells and monocytes were negatively selected from PBMCs using the EasySep® Human NK cell enrichment and EasySep® Human monocytes enrichment kits, respectively, purchased from Stem Cell Technologies (Vancouver, BC, Canada). Purified NK cells and monocytes were stained with anti-CD16 and anti-CD14, respectively, to measure purity using flow cytometric analysis. Samples showing greater than 95% purity were used for the study.

### Bacteria sonication

Probiotic bacteria, AJ2 is a combination of seven different strains of gram-positive probiotic bacteria (*Streptococcus thermophiles, Bifidobacterium longum, Bifidobacterium breve, Bifidobacterium infantis, Lactobacillus acidophilus, Lactobacillus plantarum, and Lactobacillus paracasei*) selected for their superior ability to induce optimal secretion of both pro-inflammatory and anti-inflammatory cytokines in NK cells [69]. First, the bacteria were thoroughly vortexed, sonicated on ice for 15 s, set at a 60% amplitude, and incubated for 30 s on ice. At every five pulses, a sample was observed under the microscope until at least 80 percent of the bacterial cell walls were lysed. On average, approximately 20 rounds of sonication/incubation were conducted for complete sonication. Final product sAJ2 were aliquoted and stored in −80 °C freezer. Sonicated AJ2 (sAJ2) (10 mg/1 ml) was weighed and resuspended in RPMI 1640 containing 10% FBS.

### Generation of osteoclasts and supercharged NK cells

To generate osteoclasts (OCs), monocytes were cultured in alpha-MEM media supplemented with M-CSF (25 ng/mL) and RANKL (25 ng/mL) for 21 days, the media was replenished every three days. Human purified NK cells were activated with rh-IL-2 (1000 U/ml) and anti-CD16 mAb (3 µg/ml) for 18–20 h before they were co-cultured with OCs and sAJ2 (OCs:NK:sAJ2; 1:2:4) in RPMI 1640 medium containing 10% FBS. Allogeneic healthy donor-derived OCs were used for the experiments. The medium was refreshed every three days with RPMI containing rh-IL-2 (1500 U/ml).

### Treatment of tumors with NK cells supernatants

For primary NK cells, purified NK cells were activated with rh-IL-2 (1000 U/ml) and anti-CD16 mAb (3 µg/ml) for 18–20 h before the supernatant was harvested. For sNK cells, the supernatant was harvested on day 15 post rh-IL-2 (1000 U/ml), and anti-CD16 mAb (3 µg/ml) treated NK cells were co-cultured with OCs and sAJ2. The supernatant volume of primary activated NK cells and sNK cells was determined based on the IFN-γ required and was assessed with an ELISA specific to IFN-γ. OSCSCs and MP2 were treated with an average total of 2000 pg and 5000 pg, respectively. The total amount is divided into 4 treatments for 4 days at 24 24-h intervals.

### Surface staining and cell death analysis

Staining was performed by labeling the cells with antibodies as described previously [56, 70, 71]. The percentage of dead cells was determined propidium iodine (PI) (100 μg/ml) staining using flow cytometric analysis. Flow cytometric analysis was performed using an Attune NxT flow cytometer (Thermo Fisher Scientific, Waltham, MA), and FlowJo v10.4 (BD, Oregon, USA) were used for analysis. Beckman Coulter Epics XL cytometer (Brea, CA), and results were analyzed in the FlowJo vX software (Ashland, OR).

### Enzyme-Linked Immunosorbent Assays (ELISAs) and multiplex cytokine assay

Single ELISAs were performed as previously described [56]. To analyze and obtain the cytokine and chemokine concentration, a standard curve was generated by either two- or three-fold dilutions of recombinant cytokines provided by the manufacturer. For multiple cytokine array, the levels of cytokines were examined by multiplex assay, which was conducted as described in the manufacturer’s protocol for each specified kit. Analysis was performed using a Luminex multiplex instrument (MAGPIX, Millipore, Billerica, MA), and data was analyzed using the proprietary software (xPONENT 4.2, Millipore, Billerica, MA).

### eSight

xCELLigence RTCA eSight (Agilient, USA) was purchased, and cell behavior and cell function were studied using real-time biosensor impedance-based and image-based measurements. The impedance-based xCELLigence technology utilizes proprietary microplates (E-Plates View 96) embedded with gold biosensors at the bottom of each well, which serve to non-invasively quantify cell behavior. Throughout an experiment, the biosensors monitor cell metrics such as proliferation, adhesion strength, changes in morphology, migration, and differentiation. On day 1, 50 μl of the respective media was added to each well, and the machine was run once to measure the background. Subsequently, target cells were seeded per well, and the machine was run overnight for adhesion. The impedance of each well was monitored every 15 min, and the images of the cells were acquired every hour. After incubating for 18–24 h, different concentrations of effector cells were added to each well, with a twofold dilution for each target cell type. Impedance readings were recorded at 15-min intervals, and images were captured at 60-min intervals for 48–72 h.

### ^51^Cr release cytotoxicity assay

The ^51^Cr release cytotoxicity assay was performed as previously described [72]. Briefly, different ratios effectors and ^51^Cr–labeled target cells were incubated for four hours. After which, the supernatants were harvested from each sample, and the released radioactivity was counted using the gamma counter. The percentage specific cytotoxicity was calculated as follows:

$$\% {\rm{cytotoxicity}}=\frac{{\rm{Experimental\; cpm}}-{\rm{spontaneous\; cpm}}}{{\rm{Total\; cpm}}-{\rm{spontaneous\; cpm}}}$$

LU 30/10^6^ is calculated by using the inverse of the number of effector cells needed to lyse 30% of target cells × 100.

### Statistical analysis

Prism-10 software was used for statistical analysis. An unpaired or paired, two-tailed Student’s *t*-test was performed for experiments with two groups. One-way ANOVA with a Bonferroni post-test was used to compare different groups for experiments with more than two groups. Duplicate or triplicate samples were used for assessment. The following symbols represent the levels of statistical significance within each analysis: ****(*p*-value < 0.0001), ***(*p*-value 0.0001–0.001), **(*p*-value 0.001–0.01), *(*p*-value 0.01–0.05).

## Supplementary information

Data Set 1

## Data Availability

All data generated or analyzed during this study are in this published article.
